# Case Report: a giant liposarcoma of the spermatic cord

**DOI:** 10.3389/fonc.2025.1490559

**Published:** 2025-03-26

**Authors:** Mingshan Wang, Yong Fu, Xiaowen Liu, Zheng Liu

**Affiliations:** ^1^ Department of Urology, Shandong Provincial Hospital Affiliated to Shandong First Medical University, Jinan, China; ^2^ Department of Urology, The Second Affiliated Hospital of Bengbu Medical University, Bengbu, China; ^3^ Department of Central Laboratory, Shandong Provincial Hospital Affiliated to Shandong First Medical University, Jinan, China

**Keywords:** liposarcoma of the spermatic cord, imaging, differential diagnosis, surgery, case report

## Abstract

**Background:**

Liposarcoma of the spermatic cord is an extremely rare urological malignancy, with fewer than 300 cases reported in the literature worldwide, and it is often difficult to distinguish from inguinal hernias and epididymal cysts. Typically, it presents as an asymptomatic, slow-growing paratesticular mass.

**Case presentation:**

The case described herein involves a 59-year-old man who presented to our hospital with a painless mass in the left scrotum. Physical examination revealed a fixed, firm mass in the left scrotum. Ultrasonography of the scrotum demonstrated an inhomogeneous echogenic mass measuring approximately 113 x 83 x 62 mm on the left side. Testicular MRI showed a mass in the left scrotum measuring approximately 67 x 56 x 98 mm, exhibiting isointence T1 mixed with high T2 signals. The patient then underwent surgery and pathology confirmed a liposarcoma of the spermatic cord. Currently no signs of tumor recurrence on follow-up.

**Conclusions:**

Liposarcoma of the spermatic cord is an exceedingly rare condition, for which surgical intervention is the preferred treatment option. While there is no definitive evidence supporting the use of adjuvant radiotherapy following surgery, it remains necessary in cases where surgical margins are uncertain.

## 1 Introduction

In adults, a mass within the scrotum may either be located in the testicle itself or adjacent to it. While paratesticular tumors are infrequently encountered, comprising less than 5% of scrotal masses—including those in the epididymis and spermatic cord—the paratesticular area can give rise to tumors with a wide variety and spectrum of behavior (1). Primary soft tissue sarcomas make up only 2% of all malignant tumors affecting the male genitourinary system, representing the least common malignant tumors in this region (2). About 75% of male genitourinary sarcomas arise from the spermatic cord (3). Liposarcoma originating in the spermatic cord is an exceptionally rare cancer, with fewer than 300 documented cases in global literature (4, 5). When it occurs within the spermatic cord, it can mimic conditions such as an inguinal hernia, an epididymal cyst, or spinal liposarcoma, making it difficult to recognize. Typically, it manifests as a dense, palpable paratesticular mass that is asymptomatic and grows slowly (6, 7). Five-year survival rates can vary widely, ranging from 15% to 85%, based on factors such as tumor grade, site of the tumor, and the feasibility of complete surgical resection.

There is a scarcity of information regarding liposarcoma, and currently, there are no official guidelines or recommendations available for the diagnosis, treatment, and follow-up care of these patients (8, 9). Ultrasound is used as the main imaging technique primarily due to its high sensitivity to both intratesticular and extratesticular lesions, alongside its ease of use and relatively low cost. Even though the diagnosis is confirmed in approximately 50% of cases, CT or MRI continues to play a crucial role in aiding diagnosis and planning surgery for suspected malignant lesions in the inguinal area (6, 7). The definitive diagnosis is established through histological, immunohistochemical, and cytogenetic evaluations, which are considered the gold standard. Typical histological findings of liposarcoma of the spermatic cord include sarcoma, nonfatty degeneration, and high-grade cellular components, which are mainly classified into well-differentiated, dedifferentiated, myxoid, and pleomorphic variants.

## 2 Case presentation

**Figure 1:**
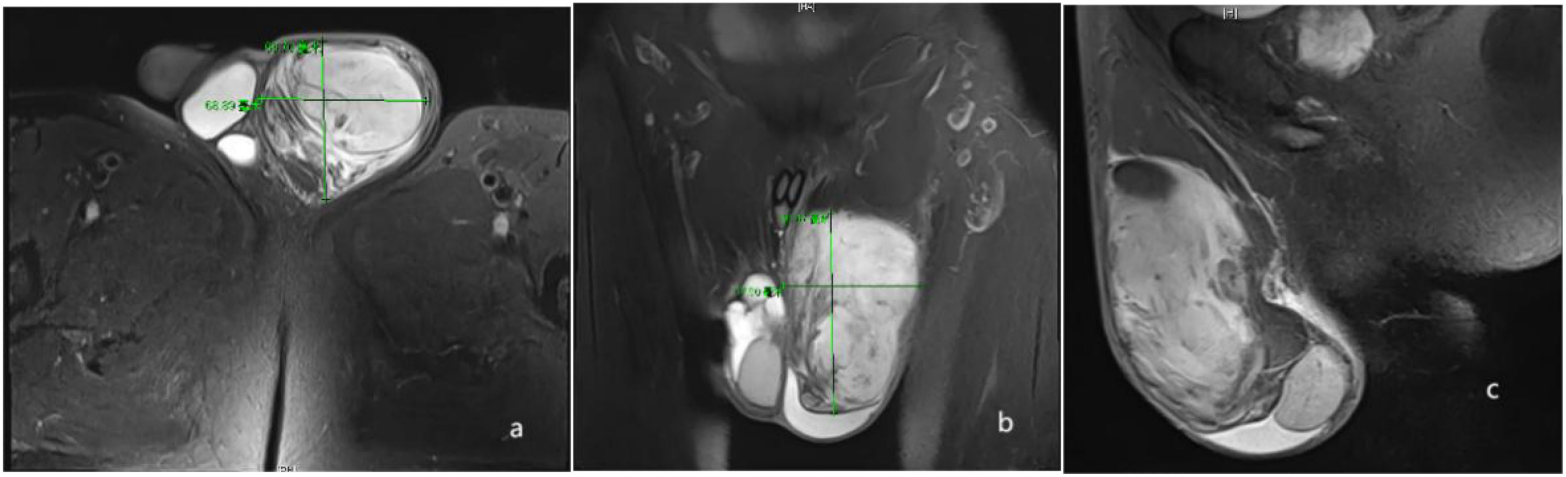
Transversal **(a)** coronal **(b)** and sagittal **(c)** MRI scan of the mass of the left spermatic cord. MRI indicated that the volume of the left scrotum was enlarged, revealing an isotropic T1 and mixed long T2 signal mass within. The borders of the mass were well-defined, measuring approximately 67 x 56 x98 mm. Additionally, multiple strips of short T2 signals were observed within the mass, accompanied by mild localized diffusion restriction on DWI and ADC maps. The left testis appeared compressed, and some pelvic adipose tissue had herniated into the scrotum through the left inguinal canal.

**Figure 2:**
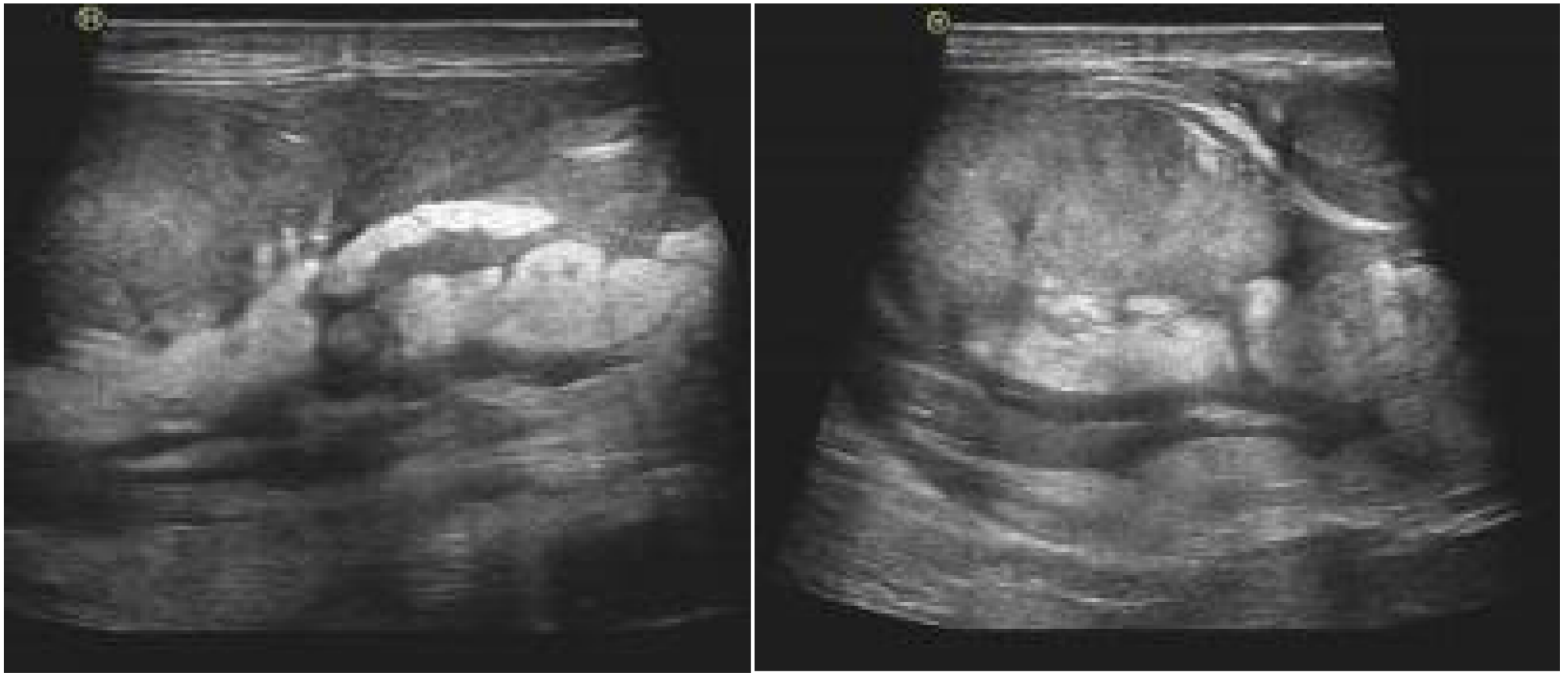
Ultrasound examination showed uneven echo quality in the sperm area above the left testicle. The mass is approximately 113 x 83 mm, with unclear limitations, irregular morphology, internal echo inequality and fat-like echoes extending to the inguinal canal.

A 59-year-old man presented with a painless left-sided scrotal mass. The patient reported that he had noticed a left scrotal swelling. No pathological abnormalities were found in the testes and epididymis. Magnetic resonance imaging (MRI) showed a 67 x 56 x 98 mm distended mass in the left spermatic cord (**Figure 1**). Magnetic resonance imaging (MRI) showed part of the pelvic adipose tissue herniated into the scrotum through the left inguinal canal, located above the scrotum, and the left testis was compressed; the right testis did not show any obvious abnormality, and no enlarged local lymph nodainless scrotal mass approximately 2 years ago, which had gradually increased in size since then. Physical examination revealed a solid mass on the left side of the scrotum with a smooth surface. Tumor markers (human chorionic gonadotropin, alpha-fetoprotein and lactate dehydrogenase) were within the reference range. Scrotal ultrasonography revealed a 11.3 x 8.3 x 6.2 mm oval (**Figure 2**), well confined, inhomogeneous es were found.

**Figure 3:**
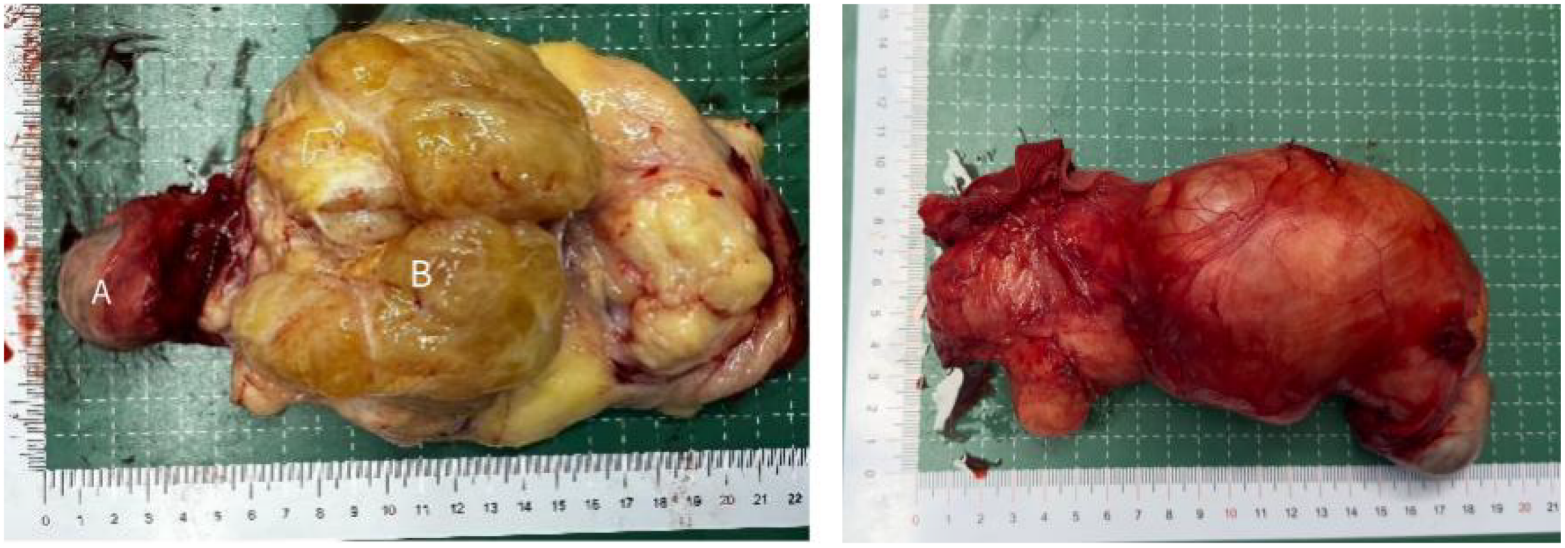
Macroscopic findings of the surgical specimen. (A: normal testis, B: tumor tissue).

**Figure 4:**
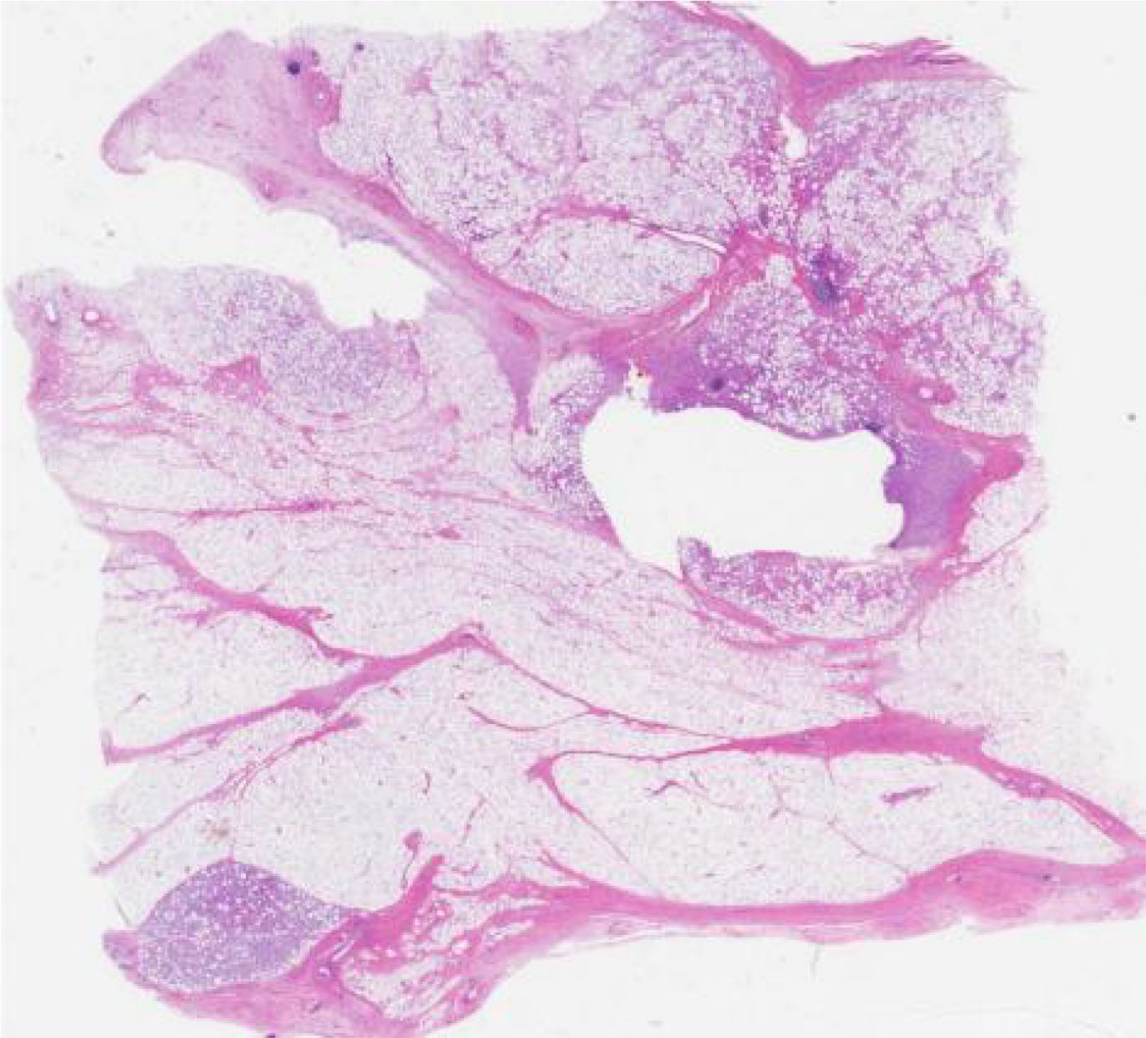
Histopathology showed spindle cell hyperplasia with mucinous changes and large nuclei with anisotropy.

Based on clinical and imaging manifestations, surgical exploration of the spermatic cord and scrotum was performed using an inguinal approach. The left spermatic cord was incised and a yellow multilobulated mass measuring 100 x 80 mm was found (**Figure 3**). The mass involved the entire circumference of the spermatic cord and travelled down into the scrotum to encircle the testis. The testis and epididymis were normal and no tumor infiltration was seen. Based on MRI and intraoperative findings, we decided to perform radical orchiectomy with high ligation of the spermatic cord. The postoperative course was uneventful and the patient was discharged on postoperative day 4. Pathological analysis showed that the tumor was composed of relatively mature adipocytes, single vesicular adipoblasts in the focal area, fibrous tissue with mucus deformation in the stroma and an intact tumor capsule (**Figure 4**). Currently, patients receive tumor follow-up with abdominal MRI and chest CT every three months to rule out tumor metastasis. The patient has given informed consent to share the information provided here.

## 3 Discussion

Liposarcoma affecting the paratesticular tissues, which include the spermatic cord or epididymis, was initially documented in 1952, and this rare tumor represents around 5% to 7% of paratesticular sarcomas (10–12). Typically, primary malignant tumors of the spermatic cord emerge below the external inguinal ring, leading to their presentation primarily as scrotal masses instead of inguinal ones. The challenge in diagnosing these tumors lies in their common presentation as painless scrotal masses; as such, liposarcomas of the spermatic cord are frequently misinterpreted as inguinal-scrotal hernias, lipomas, syringomyelia, epididymal cysts, or testicular neoplasms.

The traditional ultrasound depiction of seminomatous liposarcoma presents as a hypervascular and heterogeneous mass, featuring regions of hyperechoicity that correspond with the varying levels of fat or lipoma present within the tumor (8). While ultrasound is beneficial for assessing the mass’s size, location, and consistency, additional imaging modalities are frequently necessary, as ultrasound lacks pathological characteristics to differentiate between benign and malignant lesions. Computed tomography (CT) and magnetic resonance imaging (MRI) are more effective diagnostic imaging methods for accurately determining the tumor’s size, location, and tissue properties, along with evaluating the spermatic cord and testicular status (13–15). Typically, CT shows a mass with thickened fat, including non-lipomatous septa or soft tissue nodules (16). Given that primary spermatic cord tumors lack distinctive imaging signs or patterns, a histopathological assessment is typically essential for establishing a conclusive diagnosis (17). The 2020 WHO classification emphasizes the distinction between well-differentiated (atypical lipomatous tumor) and dedifferentiated subtypes, with the latter exhibiting higher recurrence rates and metastatic potential (18). The five-year survival rate for well-differentiated liposarcoma is as high as 85%, while the five-year survival rate for dedifferentiated liposarcoma is only 28% (19, 20). These findings underscore the necessity of precise histopathological subtyping to guide follow-up intervals and adjuvant therapy decisions.

Some studies have shown that the tumor is mainly located in the right scrotum (21). In contrast, our case report resembles the earlier review, which showed a preference for the left side (22). Surgery is the preferred treatment for liposarcoma of the spermatic cord. Liposarcoma typically disseminates through local invasion. If the diagnosis has been confirmed prior to surgery, extensive orchiectomy is recommended, along with extensive local excision and high ligation of the spermatic cord, as was done in our case (23–25). Retroperitoneal lymph node dissection is generally not recommended unless there is clear evidence of metastasis. Resection should be thorough, and scrotal resection may be contemplated for patients with malignancy to mitigate the risk of local recurrence (26). Although the chance of recurrence exists, radical orchiectomy can lead to a favorable prognosis and lower mortality rates if it results in the complete excision of negative margins. Even in instances of incomplete resection, subsequent operations aimed at extensive resection can improve disease-free survival (23). The size of the tumor and the presence of metastases at the time of diagnosis continue to be crucial indicators of disease-specific survival (27). While radical orchiectomy with high ligation remains the gold standard, recent case series suggest that testis-sparing surgery (TSS) may be feasible in select cases with localized, well-differentiated tumors and clear intraoperative frozen section margins (28). Some patients who underwent organ preservation surgery did well and showed no signs of recurrence (14, 29–31). However, such approaches require meticulous preoperative imaging and multidisciplinary collaboration to ensure oncological safety.

Although some reports indicate that radiotherapy and chemotherapy may serve as useful adjuvant treatments, their overall efficacy is limited, and there is currently no definitive cure. And due to the rarity of the disease, there are limited data on this issue and no randomized controlled studies in the literature. Coleman et al. from Memorial Sloan-Kettering Cancer Center detailed their 20-year surgical experience with seminomas involving 47 patients. Within this cohort, 21 patients (45%) received adjuvant radiation therapy, while 9 patients (19%) underwent chemotherapy. However, the researchers were unable to establish the efficacy of these therapeutic interventions. Notably, among the 21 patients who underwent reoperation with wide resection following prior incomplete resection, there was a trend toward improved disease-free survival (p = 0.059). Furthermore, when surgical margins were positive at both the first and second resections, disease-free survival was significantly shorter over time (p < 0.05), underscoring the critical importance of aggressive surgical approaches to achieve complete tumor resection (23).

Recognized treatments for liposarcoma of the spermatic cord include lumpectomy of the mass, radical orchiectomy, and high ligation of the spermatic cord. Extrapolated data from existing studies on extremity sarcomas suggest that adjuvant radiotherapy or chemotherapy may be critical in high-risk situations. For example, Morozumi et al. administered adjuvant chemotherapy to multiple recurrences and high-grade tumors and concluded that the disease was stable at 8-month follow-up (32). A phase II trial by Haas et al. (2022) demonstrated that adjuvant radiotherapy in spermatic cord sarcomas with close margins (<1 cm) reduced 5-year local recurrence rates from 38% to 12% (33). Despite our patient’s disease-free status, long-term monitoring through physical examinations and cross-sectional imaging is necessary.

This article presents a significant contribution by reporting an exceptionally rare case of liposarcoma of the spermatic cord, thereby providing a valuable reference for the clinical diagnosis and treatment of this condition. The accuracy of diagnosis and comprehensiveness of treatment were ensured through collaboration among the urology, pathology, and imaging departments. Patients were followed up for up to one year, yielding important insights into disease recurrence and prognosis. Detailed imaging and pathology images are included to enhance readers’ understanding and visualization of the case features. However, this study has limitations. Given the extreme rarity of liposarcoma of the spermatic cord, only one case is reported, which restricts the generalizability and representativeness of the results. Additionally, the rarity of the case precluded the establishment of a control group for comparative analysis, further limiting the generalizability of the findings. Although a one-year follow-up period was conducted, this duration remains insufficient for a thorough assessment of long-term prognosis and recurrence rates. Furthermore, the treatment protocol outlined in this paper may not be universally applicable to all similar cases, indicating that individualized treatment approaches require further investigation.

## 4 Conclusions

Liposarcoma of the spermatic cord is an extremely rare urologic malignancy that is easily misdiagnosed clinically as inguinal hernia or epididymal cyst. Surgical resection (radical orchiectomy combined with high spermatic cord ligation) is the treatment of choice for this disease, and its prognosis is closely related to tumor grade, resection completeness, and metastasis. Although there is no clear evidence to support routine postoperative adjuvant radiotherapy, it should still be considered when the margins of excision are uncertain. Given its high risk of recurrence and poor prognosis, patients need to undergo long-term imaging follow-up to monitor metastasis and recurrence. Clinicians should raise awareness of this disease and develop individualized treatment plans in conjunction with a thorough imaging evaluation.

## Data Availability

The original contributions presented in the study are included in the article/supplementary material. Further inquiries can be directed to the corresponding author.
